# Subjective frequency of seeking for support from different sources and mental health among juvenile convicts

**DOI:** 10.1192/j.eurpsy.2022.1618

**Published:** 2022-09-01

**Authors:** L. Shaigerova, A. Dolgikh, O. Almazova, F. Ushkov

**Affiliations:** Lomonosov Moscow State University, Psychology, Moscow, Russian Federation

**Keywords:** juvenile convicts, social support, mental health

## Abstract

**Introduction:**

As mental health and emotional wellbeing while serving a sentences contribute a lot to the successful resocialization of juvenile convicts, it is necessary to study the factors that influence them.

**Objectives:**

To consider the relationship between the subjective frequency of social support from various sources and indicators of mental health and well-being of juvenile convicts.

**Methods:**

The study used DASS (Lovibond, Lovibond, 1995), WEMWBS (Tennant et al., 2007), PANAS (Watson, Clark, Tellegen, 1988), SPANE (Diener et al., 2010), and a question to measure the subjective frequency of seeking for support from different sources (parents, close relatives, friends, mentors, psychologists, other convicts etc.), as measured on a 4-point scale from 1 (never) to 4 (constantly). 657 juvenile convicts aged 15 to 18 (M=17.0; SD=0.8) took part in the study.

**Results:**

Regression models (R>0.5) were obtained by means of regression analysis (frequencies of seeking for support from different sources were taken as independent variables). The declared frequency of seeking for support from mentors served as a predictor of psychological well-being (Beta 0.148, t=2.271; p=0.024), the level of depression (Beta=-0.193, t=-2.917; p=0.004), anxiety (Beta=-0.157, t=-2.365; p=0.018) and stress (Beta=-0.142, t=-2.136; p=0.033), as well as of negative experience (Beta=-0.202, t=-3.025; p=0.003). The declared frequency of seeking suppotr from psychologists predicted the level of positive experience (Beta=0.128, t=2.052; p=0.041) and of positive affects (Beta=0.145, t=2.259; p=0.024).

**Conclusions:**

Mental health, well-being and emotional state of juvenile convicts are directly related to the perception of the frequency of seeking support from the employees of the correctional camps (mentors and psychologists).

**Disclosure:**

No significant relationships.

